# Single cell RNA sequencing reveals hemocyte heterogeneity in *Biomphalaria glabrata*: Plasticity over diversity

**DOI:** 10.3389/fimmu.2022.956871

**Published:** 2022-09-05

**Authors:** Rémi Pichon, Silvain Pinaud, Emmanuel Vignal, Cristian Chaparro, Marine Pratlong, Anaïs Portet, David Duval, Richard Galinier, Benjamin Gourbal

**Affiliations:** ^1^ IHPE, Univ Montpellier, CNRS, IFREMER, Univ Perpignan Via Domitia, Perpignan, France; ^2^ Cambridge Institute, Li Ka Shing Center, Cancer Research UK, Cambridge, United Kingdom; ^3^ IHPE, Univ Montpellier, CNRS, IFREMER, Univ Perpignan Via Domitia, Montpellier, France; ^4^ Plateforme MGX - Montpellier GenomiX, Institut de Génomique Fonctionnelle, Montpellier, France; ^5^ Molecular Immunity Unit, Department of Medicine, Medical Research Council (MRC) Laboratory of Molecular Biology, University of Cambridge, Cambridge, United Kingdom

**Keywords:** single cell RNA seq, *B. glabrata*, hemocytes, innate immune system, *S. mansoni*

## Abstract

The freshwater snail *Biomphalaria glabrata* is an intermediate host of *Schistosoma mansoni*, the agent of human intestinal schistosomiasis. However, much is to be discovered about its innate immune system that appears as a complex black box, in which the immune cells (called hemocytes) play a major role in both cellular and humoral response towards pathogens. Until now, hemocyte classification has been based exclusively on cell morphology and ultrastructural description and depending on the authors considered from 2 to 5 hemocyte populations have been described. In this study, we proposed to evaluate the hemocyte heterogeneity at the transcriptomic level. To accomplish this objective, we used single cell RNA sequencing (scRNAseq) technology coupled to a droplet-based system to separate hemocytes and analyze their transcriptome at a unique cell level in naive *Biomphalaria glabrata* snails. We were able to demonstrate the presence of 7 hemocyte transcriptomic populations defined by the expression of specific marker genes. As a result, scRNAseq approach showed a high heterogeneity within hemocytes, but provides a detailed description of the different hemocyte transcriptomic populations in *B. glabrata* supported by distinct cellular functions and lineage trajectory. As a main result, scRNAseq revealed the 3 main population as a super-group of hemocyte diversity but, on the contrary, a great hemocytes plasticity with a probable capacity of hemocytes to engage to different activation pathways. This work opens a new field of research to understand the role of hemocytes particularly in response to pathogens, and towards *S. mansoni* parasites.

## Introduction

In invertebrates, innate immune response is mainly carried out by the hemocytes, appearing as a complex family of cells specialized in immunity (1). These cells carry both the so-called cellular response of the innate immune system, through encapsulation and phagocytosis of pathogens. But also, part of the humoral response by secreting in the hemolymph many cytolytic/cytotoxic compounds like antimicrobial factors, oxygen/nitrogen reactive species (ROS/NOS), proteases or toxins (2–4).

Despite the important role played by hemocytes in the invertebrates’ immune response, these cells are still very poorly characterized. Different types of invertebrate hemocytes have been described in the literature (5), mainly according to their differential abilities to respond to pathogens, from phagocytosis, cell to cell adhesion or degranulation of humoral factors (1), but also based on their morphological features (shape, size, intracytoplasmic granules, vacuoles). However, hemocyte morphology studied alone is not sufficient to define a clear functional classification of hemocytes. Indeed, a direct relationship between hemocyte morphology and their specific immunological functions against pathogens is quite impossible to establish. However, the development of new technology of sequencing at the single cell level [single cell transcriptomics (scRNAseq) (6)] is now available to access the invertebrate hemocyte functional diversity.

Single-cell transcriptomics approaches have been firstly, and successfully developed in vertebrate models to describe the high specialization of innate immune cells and study their heterogeneities (7–9). Then, this technology was successfully transferred to various other non-vertebrate models, especially for infectious diseases such as plasmodium sp., the agent responsible for malaria disease Thousands of cells across the different parasite differentiation stages were sequenced to produce the very first parasite developmental cell atlas (10, 11). Such a tool allows the definition of new-targeted therapeutic strategies and the understanding of the mechanisms of transmission to its hosts. The study of metazoan parasite species, such as *Schistosoma mansoni*, the agent responsible for human intestinal Schistosomiasis has also benefits from this technology. The scRNA seq approaches on stem cell populations from juvenile *Schistosoma* allowed to precisely define 9 transcriptomic populations of somatic cells, and the characterization of conserved gene sets involved in the regulation of germline cells (12). This technology has proven to be a major asset also in the description of hemocyte populations in invertebrate hosts, improving the definition of the heterogeneity of immune cell populations in insects such as Drosophila, mosquitoes and silkworm (13, 14) or crustacean shrimp (15–19). ScRNA seq has allowed redefining, for the silkworms, the complexity of hemocyte populations in this model and the impact of a baculovirus infection (*Bombyx mori nucleopolyhedrovirus*) on some hemocyte populations (20). In addition, for mosquitoes, hemocyte complexity following infection by *Plasmodium* has been characterized, with the definition of 6 transcriptomic clusters in comparison to the 3 populations historically described based on morphological traits (21). These scRNAseq approaches have been shown to be very efficient in all these invertebrate host/parasite models and demonstrate the feasibility of this technique to decipher the complexity of hemocyte cell populations as well as to understand the immunological mechanisms activated in hemocytes in response to pathogens.

In the present study we paid a particular attention to a schistosomiasis vector snail, the gastropod *Biomphalaria glabrata*. Schistosomiasis remains today the second human parasitic disease after Malaria, in terms of morbidity and mortality in endemic areas (22), mainly South-America and sub-Saharan Africa. Schistosomiasis disease is caused by a flatworm of the genus *Schistosoma* which uses the gastropod *Biomphalaria* as an obligatory intermediate host to complete its life cycle. Thus, the comprehension of the immunological interactions between *B. glabrata* and *S. mansoni* would help in developing new strategies to fight or control this disease. Many studies to date have focused on these molecular interactions (23), and research has rapidly identified the key role played by hemocytes in recognition and/or killing of the parasite (3). However, despite their potential importance, knowledge on *Biomphalaria* hemocytes remains very sparse and a clear description of morphological populations or subpopulations, their proportion, and their functions are still a matter of debate (24).

Among those studies on the description of hemocyte populations in *Biomphalaria*, the morphological description of the cells by optical microscopy coupled with lectin surface labeling highlighted at least four hemocyte populations (25). Currently, some works describe two populations, hyalinocytes and granulocytes (26, 27) using a combination of light and electron transmission microscopy as well as flow cytometry approaches. Other results using optical and electron microscopy (24), describe five morphologically distinct populations of hemocytes: hyalinocytes (that can be split into three subpopulations of hyalinocytes I, II and III), granulocytes and blast-like cells. However, despite these morphological characteristics, it is still complex to define their specific biological functions. It has been demonstrated that some morphologically similar cells display different biological functions or that distinct hemocyte populations display similar functions. For example, *Biomphalaria glabrata* hyalinocytes and granulocytes are known to be involved directly in the cellular response through non-self-recognition, phagocytosis and encapsulation (25). In this context, a recent study (28) has attempted to describe the function of specific hemocyte populations using a serial dilution method to isolate granulocytes and hyalinocytes and described more accurately the specific functions associated with these cells using a massive transcriptomic sequencing approach. Finally, it has been demonstrated for blast-like cells, often considered as prohemocytes (undifferentiated cells) (24), that differential gene expression patterns could be observed, indeed some subpopulations of blast-like cells produce for example a complement like factor, named BgTEP1 (29, 30), acting as an opsonin molecule involved in the immune response against *S. mansoni* parasite, demonstrating an unexpected level of cellular complexity yet indistinguishable morphologically.

This is why, herein we used a droplet-based system of single cell RNA sequencing (31) to describe without *a priori* and for the first time, the level of heterogeneity and diversity of hemocyte populations in the freshwater snail *B. glabrata.* We discovered a slight diversity of transcriptomic populations defined by sets of marker genes specific to each of the populations combined with cell lineage relationship between different hemocyte subpopulations. These transcriptomic populations are still rather complex to correlate with the published morphological populations from microscopy, flow cytometry and label-free proteomic analyses, mostly because of the shallowness of the *Biomphalaria glabrata* genome/transcriptome/proteome annotation. In addition to bringing new clues of rather hemocyte plasticity than diversity in *Biomphalaria glabrata*, we proposed and discussed herein that complementary approaches must be used to eventually define hemocyte populations and start to face hemocyte biology where it matters most.

## Materials and methods

### Biological sample experiments

We used a *Biomphalaria glabrata* strain originated from Recife locality in Brazil (BgBRE2), recovered in 1975 and maintained since then under constant laboratory conditions. Snails were maintained at 26°C in glass aquaria and fed with green leaf lettuce *ad libitum*.

### Hemocytes preparation for morphological characterization

10 snails (size: 8-10 mm) were retained to collect hemocytes by puncturing the hemolymph (30µL per snail) according to a widely used protocol previously established using the defence reflex of hemolymph released from the cephalo-pedal sinus when the snail shrinks in its shell (32). Hemolymph was transferred on a polystyrene slide (Caplugs evergreen) and stood in a humid chamber for 30 min for hemocytes to adhere to the slide. Then, hemocytes were stained with a panoptic May-Grünwald Giemsa (MGG) type staining method by MCDh (RAL Diagnostics) following the manufacturer recommendations. Briefly, hemocyte slides were sunk during 6 min in MCDh1 solution, 1min in a first bath of MCDh2, 2 min in a second bath of MCDh2, 1min in MCDh3, then 10 seconds in MCDh4. Slides were dried and mounted with Dako^®^ fluorescent mounting medium (Agilent, S3023) and finally the hemocyte populations and their proportions were counted under light microscopy using a 100X objective (**Supplementary Table 1**).

### Hemocytes preparation for droplet scRNA sequencing

Pooled hemolymph (500µl) from 50 snails was recovered into 2ml tubes and mixed with 1.5ml of modified anticoagulant solution (98mM NaOH, 186mM NaCl, 1.7mM EDTA, 1.7mM citric acid) (13). Sample was passed through a 30µm pre-separation filter (Miltenyi Biotec) to eliminate cell aggregates and obtain a suspension of unique cells. Then hemocytes were counted using a Malassez chamber and cell viability was measured with trypan blue exclusion technique. Samples were then spin-down (2700g, 5min, 4°C) to pellet the hemocytes and re-suspended in 50µl of anticoagulant solution [30% of BGE medium (33)] and 70% anticoagulant modified solution). To obtain a concentration of 1000 cells per microliter. Samples were then processed by MGX platform (IGH Montpellier, France) for scRNA droplet isolation (Chromium, 10X genomics) and RNA sequencing.

### Single-cell RNA sequencing and data processing

Single cell processing procedure is defined by the MGX platform (Montpellier GenomiX). Single cell suspension was obtained by a Chromium Single-Cell Controller (10X Genomics). Library preparation was performed with Single Cell 3’ Reagent kits V3.1 (10X Genomics) using 10x Next GEM Technology barcode and validated by DNA quantification with Fragment Analyzer (kit High Sensitivity NGS) and qPCR (ROCHE LightCycler 480). Libraries were sequenced with an illumina NovaSeq 6000 (Illumina) and SBS (Sequence By Synthesis) techniques using NovaSeq Reagent kits (100 cycles). Output results and matrix generation were processed with 10X Genomics Cell ranger v3.1.0 software (http://10xgenomics.com).

All available mitochondrial gene sequences were recovered from the NCBI database (34, 35) and gene names corresponding to these sequences (**Supplementary Table 2**) were retrieved using blastn (36) on the *Biomphalaria glabrata* genome annotation version 1.6 available on Vector Base website (https://vectorbase.org/vectorbase/app/record/dataset/TMPTX_bglaBB02, 15/10/2021) (37).

### Cell clustering and genes expression

Filtered matrix sorted by Cell Ranger (10X Genomics Cell Ranger 3.1.0) was used and analyzed on R (Version 4.1.0 (2021–05–18)) using Seurat package [Version 4.0.4 (38)]. Hemocytes with more than 50 unique expressed genes were kept and genes expressed in 3 or more cells were retained for the generation of the gene-cell data matrix and downstream analysis. Low quality cells were filtered out excluding cells with less than 750 or more than 3100 unique expressed genes and cells with a proportion of mitochondrial transcript higher than 5% were excluded from the analysis. Log-normalized method given by the Seurat package was used to normalize the data. Thereafter, 2.000 highly variable genes were obtained using FindVariableFeatures function, these genes were scaled and used to perform Principal Component Analysis (PCA). JackStrawPlot and ElbowPlot functions were used to determine the top principal component (15:25) that is most representative of the data set for each reduction. The dataset clustering was conducted by the FindClusters function according to identified PCs. Visualization in two dimensions of the clustering was made through the use of non-linear dimensional reduction, UMAP (Uniform Manifold Approximation and Projection for dimension reduction) and tSNE (t-distributed Stochastic Neighbor Embedding) projection. We used Seurat (FindAllMarkers option using wilcoxon rank sum test for each cluster) to determine differentially expressed genes and gene markers (**Supplementary Table 2**) for each cluster (Log2FC>0.25 and min.pct > 0.25) and created a heatmap to represent the top n marker genes defining each cluster. Cluster-specific marker genes were determined by selecting shared characteristics as being differentially overexpressed with Log2FC > 1, expressed by a majority of cells (>80%) within the cluster and a minority of cells (<10%) in other clusters. All these marker genes were used to perform a GO enrichment analysis (fisher exact test, P-value > 0.01) using Blast2GO omicsbox (39) on *Biomphalaria glabrata* reference genome V1.6.

### Cell trajectory analysis

The cell lineage analysis was performed with bioinformatic tools Monocle3 (40) and slingshot (41) on the data processed by the Seurat package mentioned above after conversion to SingleCellExperiment object. Some transcriptomic clusters considered as being the most distant and different with respect to the marker genes that define them were removed from the analysis to perform this cell lineage. To identify temporally expressed genes we used a general additive model (GAM) based on bioinformatic workshop (42). R script used for all the bioinformatic analysis done with Seurat and Monocle3 packages were available on Zenodo (accession #: 10.5281/zenodo.6951346).

### Flow cytometry, cells sorting and Label-Free proteomic sequencing

Pooled hemolymph of 300 BgBRE snails was used for cytometry cell sorting. Briefly, hemolymph was extracted from the snails as previously described and immediately used for flow cytometry and cell sorting, 25 μL of hemolymph was recovered from each snail for a total of 3.5 ml. Cell sorting was performed, using a FACS Canto from BD Biosciences (RIO Imaging Platform, Montpellier, France), according to the FSC and SSC parameters to discriminate each hemocyte population according to their size and granularity. With the help of FACSDIVA software, four gates were defined based on different SSC and FSC parameters (low SSC/FSC to high SSC/FSC). Hits detected in these gates were sorted, recovered on microscopic slides and observed under a light microscope to confirm the enrichment of different morphological hemocyte populations associated with the different settings of cell sorting gates. Finally, sorted hemocyte samples were recovered for label-free proteomic analysis. Recovered hemocytes were lysed in hypotonic buffer, protein extracts were quantified (2D-Quant kit protein quantification) and 200 µg of proteins were solubilized in laemmli buffer 4x (Biorad, hercules Califonia, USA), boiled at 95-100°C for 5 min, frozen at -80°C and send for label free sequencing to EDyP service facilities (INSERM, CEA, Grenoble, France). Protein preparation and mass spectrometry-based proteomic analyses were conducted as described in (43, 44). Protocol repeated, hereafter, from Pinaud et al., 2019. Briefly, extracted proteins were stacked in the top of an SDS-PAGE gel (NuPAGE 4 to 12%; Invitrogen) before in-gel digestion was performed using trypsin (sequencing grade; Promega). Resulting peptides were analyzed in duplicate by online nanoscale liquid chromatography tandem mass spectrometry (nanoLC-MS/MS) (UltiMate 3000 and LTQ-Orbitrap Velos Pro; Thermo Scientific) using a 120-min gradient. Peptides and proteins were identified using Mascot software (Matrix Science) and confronted against either, Uniprot database and translated genome of *Biomphalaria glabrata* snail (available at vector base: https://vectorbase.org/vectorbase/app). Qualitative differences between samples were considered as potential markers of sorted hemocyte populations and used to infer scRNAseq clusters to a potential morphological population of hemocytes.

## Results

### Morphological description of *Biomphalaria glabrata* hemocytes

**Figure 1:**
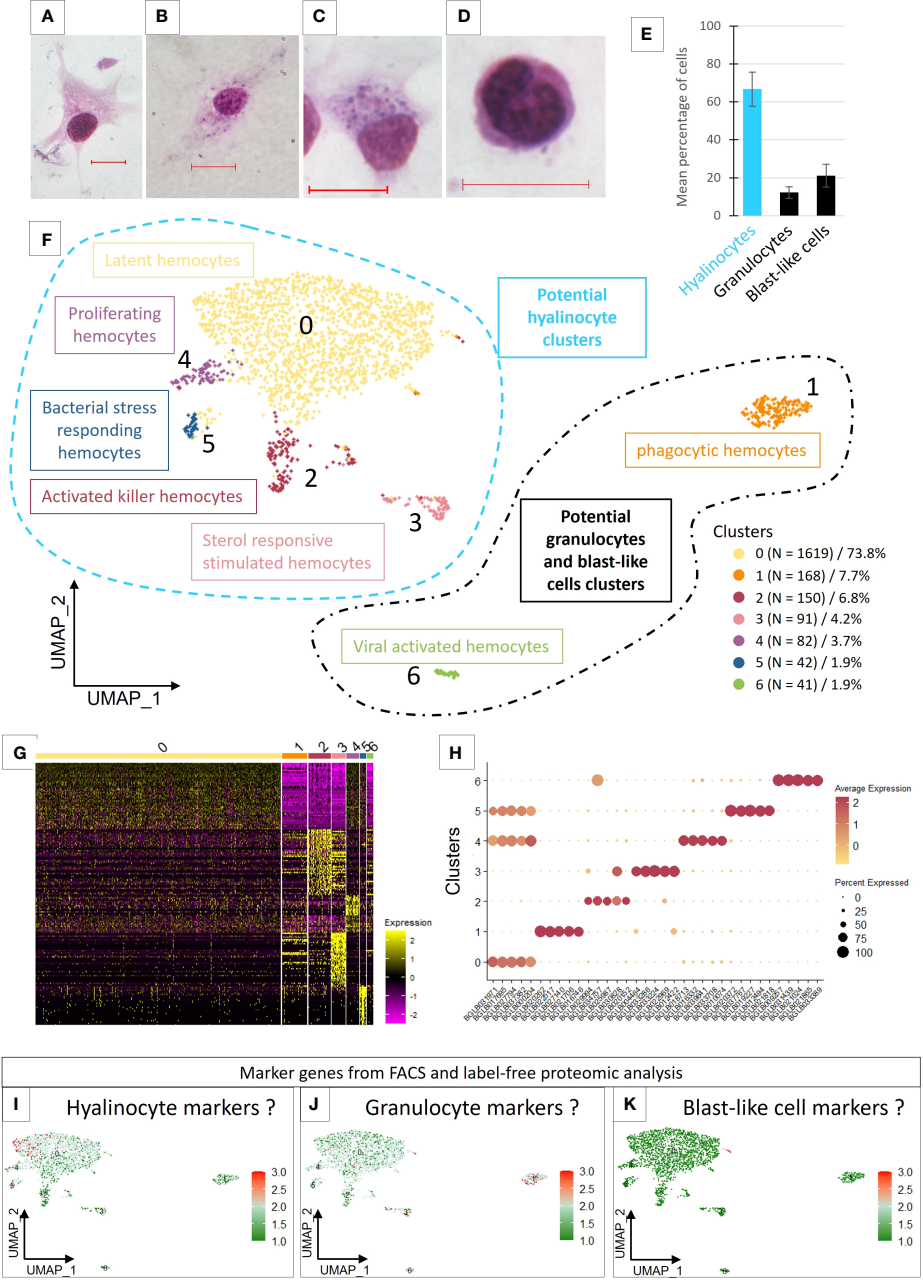
**(A–D)** Light microscopy observation of MCDh-stained hemocytes. Red bars 10µm. **(A)** hyalinocyte; **(B, C)** granulocytes; **(D)** blast-like cell. **(E)** Average number of hemocytes (1844 cells total) from 10 naive individuals according to three morphological types. **(F)** Uniform Manifold Approximation and Projection for Dimension Reduction (UMAP) plot of the 7 hemocyte clusters identified in *B glabrata* by scRNA-seq and their respective count of the cell number present in each transcriptomic cluster. **(G)** Heatmap representing the differential expression of the top 50 marker genes defined for each of the 7 transcriptomic clusters. **(H)** Dotplot representing the average expression of the 5 genes defined as markers for each cluster according to the percentage of cells expressing these genes per cluster. **(I–K)** represent the average expression levels of the genes defined as markers by the flow cytometry approach coupled with label free proteomics. Dotplot and UMAP representation of marker genes average expression of hyalinocyte **(I)**, Granulocyte **(J)** and blast-like cells **(K)**.

There is no real consensus on the morphological characterisation of hemocyte diversity for *Biomphalaria glabrata*. To clarify this purpose, we decided to establish an “hemocytogram” of circulating *B. glabrata* hemocytes after plating. Three distinct hemocyte populations were identified: hyalinocytes, blast-like cells and granulocytes (**Figures 1A–D**). The hyalinocytes are large cells (size around 35 µm) with a large nucleus, characterized by different chromatin states (with a dense purple heterochromatin and a pale purple euchromatin) (**Figure 1A**). In their cytoplasm can be observed large unstained vacuoles. These cells are characterized by a great capacity of adherence to plastic slides and by their capacity to produce large and long pseudopodia (**Figure 1A**). They represented the main quantity of circulating hemocytes in *Biomphalaria glabrata*, 66.7% +/- 9% (**Figure 1E**). The blast-like cells (**Figure 1D**), represented 21% +/- 6% of circulating hemocytes (**Figure 1E**), they were small cells (around 8µm), displaying a high nucleocytoplasmic ratio with very low adhesion capacities. These cells appear with a rounded shape even after plating on plastic slides (**Figure 1D**). Nuclei appeared very dense upon MCDh staining with a dark purple color (**Figure 1D**) indicating potentially a low level of gene transcription, these cells being often considered as pro-hemocytes. Finally, the third type of hemocytes were the granulocytes (**Figures 1B, C**) that represented 12.3% +/- 3% of circulating hemocytes (**Figure 1E**), and were not able to adhere efficiently to plastic slides. Very few cells were able to form small filopodia and were mainly characterized by a spherical shape (**Figure 1C**). Granulocytes have a dense dark purple nucleus and always show neutrophilic (deep purple) to basophilic (dark blue) granules in their cytoplasm. However, among the population of granulocytes, we were able to distinguish some morphological differences. Some granulocytes were able to adhere efficiently to the slide and form long pseudopodia that could make them difficult to distinguish from hyalinocytes (**Figure 1B**), except that these cells possessed intracytoplasmic granulations, which make them belonging to granulocytes. The proportion of these different cell populations in hemolymph remain in agreement with the data available in the literature (24) although the sizing of these cells on plastic slides appeared very hazardous due to their variable adhesion capacities, the plasticity of their morpho-anatomic parameters, and their important size and shape variability. Thus, we were not able to define clearly, (i) the exact number of hemocyte populations and (ii) the delimitation between hemocyte subtypes. Facing such limitations, we proposed to use a scRNA-seq approach to decipher the hemocyte populations and their potential role or function using their transcriptional activity and gene expression patterns rather than their morphological parameters.

### Identification of 7 distinct clusters in *B. glabrata* hemocytes by scRNA-seq

To examine hemocyte heterogeneity by scRNA-seq, isolated hemocytes from a pool of 50 naive snails were subjected to droplet scRNAseq. Approximately 3000 cells were captured using the 10X Genomics Chromium microfluidic technique and submitted to RNA sequencing. After an initial process by CellRanger (10x Genomics Cell Ranger 3.1.0), the sequencing data were aligned to the *B. glabrata* reference genome (90.1% reads mapped to the genome). We obtain 2624 estimated cells with a median gene per cell corresponding to 1885 genes among the 19 820 total genes detected and a sequencing saturation at 66%. To retain high quality single cell RNA-seq data, we removed low-quality cells with gene number less than 750 and mitochondrial genes less than 5%. Seurat R package (Version 4.0.4) was used for data processing. Following such quality control, 2193 high-quality cells were used for further analysis. For each cell, an average number of 2470 genes expressed were obtained and 7 transcriptomic clusters were identified (**Figure 1F**). Regarding read coverage by clusters, cells in clusters 0, 2, 4 and 5 have a higher number of reads compared to cells in clusters 3, 1 and 6 (**Supplementary Figure 1**). These last 3 clusters may represent cells with low transcriptional activity or small cells like blast-like cells.

Most of the hemocytes gathered within a main cluster (cluster 0) which represented 73.8% of the total cells. The other clusters (cluster 1 to 6) represented from 7.7% (cluster 1) to 1.9% of the cells respectively (**Figure 1F**). We were able to define transcriptomic profiles and specific marker genes for each cluster (**Figures 1G, H**). Cluster 0 does not have genes that are completely specific to it and appears to share several defined marker genes with the clusters 4 and 5 (**Figure 1H**). Some marker genes of the Cluster 2 are also overlapping with cells of cluster 3, but the latter possess a very specific list of marker genes.

### Gene expression signatures and candidate markers for each hemocyte cluster

Annotation and understanding of *Biomphalaria glabrata* genome/transcriptome/proteome are still very sparse. Amongst the 31 985 defined genes for about 44 406 transcripts, less than the half possess at least a protein domain information, making every enrichment or functional analysis conditioned by annotation bias. Also, it exists a redundancy in *B. glabrata* gene annotation, meaning that same gene annotation or transcripts can have different gene name/position and different transcripts. Knowing such limits but in order to create a transcriptomic identity card for the different clusters, we were interested in the function of the marker genes detected with Seurat (**Supplementary Table 2**). When possible (sufficient number of genes), we performed a Gene Ontology enrichment analyses with the blast2GO tool (**Supplementary Table 3**), taking into account only the marker genes present in at least 80% of the hemocytes from the cluster considered.

Analysis of the enriched functions (**Supplementary Table 3**) showed that hemocytes from Cluster 0 expressed genes mostly related to metabolism, energy production and protein synthesis but also functions involved in actin and cytoskeleton reorganization. These cells probably have an important translational activity but no specific specialization. We have in this cluster active cells, with generalist functions of cell maintenance but also cells able to express marker genes already described in the literature. Indeed, we found however several Pattern Recognition Receptors (PRRs) that can recognize different types of pathogens, and some of which have a very important role in immunity against *S. mansoni* parasite and especially in the recognition of this parasite, such as FREPs (45), MAM and LDL-receptor class A domain-containing 1-like (46) or genes containing von Willebrand factor EGF and pentraxin domain-containing 1 (47) that have been described in other models and which could have a role in suppressing or modulating the immunity (**Supplementary Table 2**). We proposed that cluster 0, named latent hemocytes (**Figure 1F**), is composed of circulating hemocytes that are waiting for activation in the case of a potential encounter and infection with a pathogen or the activation of alarmins. Moreover, some of the marker genes in this cluster are also expressed in clusters 4 and 5, which might suggest a strong cell filiation between these three clusters.

The five marker genes selected to characterize cluster 4 (**Supplementary Table 2**), were all genes involved in functions related to DNA replication and cell proliferation. Enrichment analyses confirm these functions (**Supplementary Table 3**). The cells in this cluster represent only 3.7% of the total number of cells (**Figure 1E**) considered in the analysis and may correspond to proliferating cells. Indeed, proliferation of circulating hemocytes has been yet demonstrated in naive *B. glabrata* snails with a proportion of 1.8% of hemocytes in proliferation (47), but no references has mentioned a specific hemocyte population capable of proliferating. These cells, considered as proliferating hemocytes (**Figure 1F**), thus appear to derive from cluster 0 and to be proliferating in response to an unknown stimulation or for maintaining a basal hemocyte turn-over.

Cluster 5 is defined by a few but specific marker genes with strong differential expression (log2FC) for the selected genes compared to all remaining cells (**Supplementary Table 2**). Most of these genes are not annotated. Some could have functions related to lipid metabolism (ganglioside GM2 activator) or being involved in response to pathogens (antimicrobial peptides, holotricin-3-like, PRRs),. This cluster could correspond to bacterial stress responding hemocytes (**Figure 1F**).

Concerning cluster 3, the enriched functions (**Supplementary Table 3**) are mainly related to the cellular component of gene ontology. The enriched functions of this cluster tend to show active cells able to set up vacuolization and degradation processes by the lysosome. Thus, many cathepsins (L1-like, Z, B), G-type lectins, cystatin-B (48) are involved in these functions. At least 7 genes encoding proteases, cathepsins (B, Z and D) and cathepsin like (L1) are represented as marker genes in this cluster and one cathepsin Z (BGLB004464) is specific to this cluster (**Supplementary Table 2**). Some of these molecules are known in the *B. glabrata*/*S. mansoni* model to have a possible role in the interaction with the parasite (49). We also find in this cluster functions involved in sterol and cholesterol metabolism, shown to have a role in modulating some immune pathways (50). This reinforces the idea that this cluster is a cluster of activated cells probably responding to a circulating factor and using sterols to respond, that is why we named this cluster sterol responsive stimulated hemocytes (**Figure 1F**).

In cluster 2, cells express some genes involved in immunity such as several cathepsins (L1-like, B, D, Z) as we can find in cluster 3 but with lower differential expression (**Supplementary Table 2**). This cluster is mainly marked by C-type lectin, peptidoglycan-recognition SC2-like or an apolipophorins-like isoform X1 which is initially known for lipid transport but which may also have a role in the immune response and especially in encapsulation (23, 51). Other genes are strongly involved in different signaling pathways (MAPK, ERBB2, etc.) or in the regulation of cell necrosis and vacuole formation (**Supplementary Table 3**). The cells that make up this cluster could therefore be activated killer hemocytes (**Figure 1F**), either dying or degrading possible phagocytosed pathogens.

Cluster 1 represents 7.7% of the total cells (**Figure 1E**) in the analysis and is marked mainly by functions involved in the activation of the immune response and different pathways related to cell phagocytosis capabilities as well as functions involved in vacuolization and cytoskeleton rearrangement processes (**Supplementary Table 3**). Among the genes selected as potential marker genes for this cluster (**Supplementary Table 2**), we also find immune genes such as a fibrinogen-related protein J1 precursor and a cathepsin B-like. Thus, this cluster could be composed of activated cells possessing phagocytosis capabilities and as described with a smaller RNA content would be strongly considered as a blast-like cell cluster.

Cluster 6 expressed genes involved in cell differentiation and migration functions (ERBB2 signaling pathway) as well as genes having immune-related annotations like cathepsin and apolipophorins, an immune receptor role like toll-like receptor or macrophage mannose receptor or even functions annotated to be involved in response to virus (**Supplementary Table 3**). This cluster is also marked by a high ribosomal activity marked by rRNA metabolic process and ribosome biogenesis functions and therefore probably a high translational activity. They are therefore cells with membrane and vesicular activity accompanied by modifications of the cytoskeleton, which could correspond once again to active phagocytic cells, may be in response to virus infection. We named this cluster viral activated hemocytes (**Figure 1F**).

### Label free proteomic from FFC/SSC-sorted hemocyte populations

Preliminary analyses of cell sorting by flow cytometry were coupled with label free proteomic analysis to attempt to associate morphology and final gene products with the objective to find specific markers of hemocyte populations. We can notice that the distribution of hemocytes according to the size and granulometry forms a continuum and does not allow to discriminate with accuracy one or more cell types or populations based on such cytometry characteristics (**Supplementary Figure 2**). Compared to a cell description on a microscopy slide where the hemocytes have specific adhesion capacities for each population, the hemocytes sorted by cytometry no longer have these characteristics. The cells are in suspension and therefore probably keep a round shape which prevents a simple discrimination by size and granulometry. However, the classical three morphological populations (hyalinocyte, granulocyte and blast-like cells) were attempted to be enriched by sorting in each of the gates (P1 to P4) (**Supplementary Figure 2**). Each of these gates corresponds to a specific FFC/SSC ratio gradient. The P4 fraction corresponds to cells defined as small to medium size with medium granularity. This fraction may allow an enrichment in hyalinocytes. The P3 fraction corresponds to cells of medium size and medium granulometry, which may allow the enrichment of blast-like cells but also some hyalinocytes. The P2 fraction corresponds to cells of small size and high granulometry, which did not give any enrichment of cells and was mostly composed of cell debris. This fraction was therefore not retained for the proteomic analyses. Finally, the P1 fraction corresponds to large size cells with high granulometry, which corresponds to a sample potentially enriched in granulocytes. The label-free proteomic analyses produce, for each of these sorted cell populations, the protein enrichment (**Supplementary Table 4**). The average expression of the genes encoding these proteins are used in the scRNA seq analysis to produce an expression mapping among the 7 clusters already described.

Unfortunately, the protein enrichment and the gene analysis from the identified proteins is not sufficient to obtain specific markers that fit with scRNAseq clustering. Indeed, all clusters seem to express all genes determined from the label free analysis. However, slight differences in the expression of these genes in some clusters are highlighted. First, we can see that few genes are specific to the fraction corresponding to an enrichment in blast-like cells. These genes are found to be carried by cells belonging to cluster 1 even though these cells seem to be closer to cluster 0 in the UMAP representation (**Figure 1K**). Genes representing the hyalinocyte-enriched fraction would be expressed by cells from clusters 0, 3 and 4 (**Figure 1I**) whereas clusters 1, 3 and 6 would represent cells expressing genes attributed to the granulocyte-enriched fraction (**Figure 1J**).

Overall results lead us to consider that cluster 0 may contain all the known cell types, since we have in this cluster the expression of all the genes that are supposed to define our enriched fractions in the three different hemocyte morphological populations. Only markers expressed specifically by clusters 1 and 6 seem belonging to the fraction enriched in granulocytes. These two clusters are also very particular because they are very different in this analysis from all other clusters and express their own marker genes. The lineage analyses do not allow us to establish a link between these clusters and the other clusters, which prevents us from studying in more detail the links that may exist between all the other clusters and cluster 0. This is why we decided to subset out cluster 1 and 6 for further analyses.

### Cell lineage

**Figure 2:**
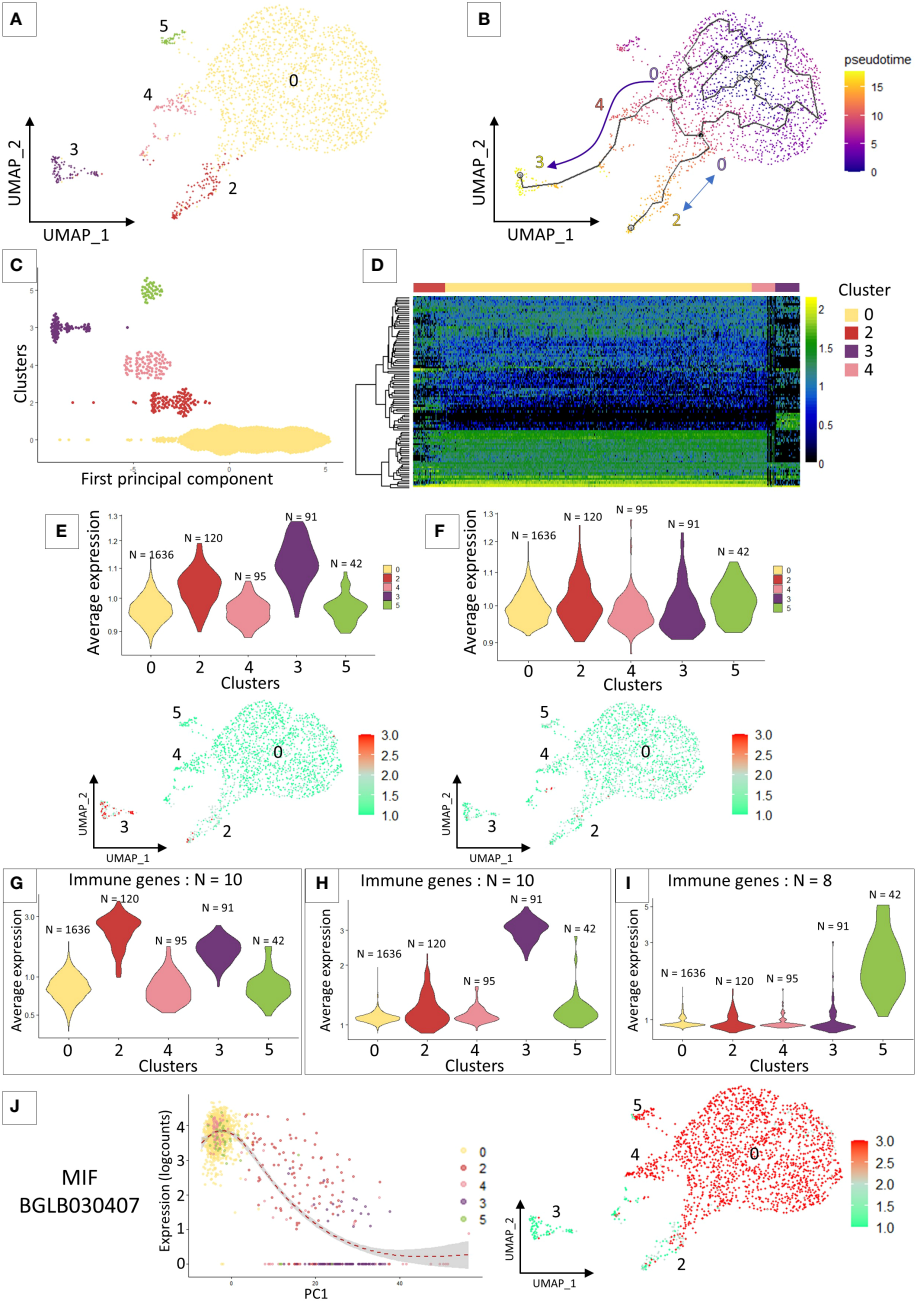
**(A)** UMAP representation of cluster 5 identified after removing clusters 1 and 6 from the previous analysis and performing reclustering. **(B)** Pseudo time analysis on the UMAP representation. Arrows represent the direction of the possible differentiation process between the different transcriptomic clusters. **(C)** Dotplot of the cluster cells ordered along the pseudo time. **(D)** heatmap representing the expression of the 100 most variable genes that characterized the pseudo-time analysis across the 4 clusters. **(E)** and **(F)** VlnPlot and UMAP representation of the average expression of marker genes from the literature. **(E)** represents granulocyte marker genes and **(F)** represents hyalinocyte marker genes. **(G, H)** and **(I)** Average expression of an immune gene set belonging to different “activated” clusters such as cluster 2 **(G)**, cluster 3 **(H)** and cluster 5 **(I)**. **(J)** Expression of the MIF gene along the pseudo-time (left graph) and the representation of its expression by the different cells of the analysis on the UMAP representation (right graph).

The cell lineage analysis on all the 7 transcriptomic clusters was inconclusive. The most differentiated clusters in the analysis (clusters 1 and 6) prevent from defining relationships between cells in the other clusters. For this reason, we removed clusters 1 and 6 and re-run the cell lineage clustering analyses (**Figure 2A**). We identified 2 particular cell lineages (**Figure 2B**), both probably originating from cluster 0 chosen by default as the starting point of the pseudo-time trajectory construction. The first lineage path connects clusters 0, 4 and 3 and the second lineage connects clusters 0 and 2 (**Figure 2B**). Cluster 5 is not linked by the pseudotime analysis to the other clusters because it shares too many similarities in terms of expressed marker genes with cluster 0 (**Figure 2C**) and the probable lack of a sufficient number of cells for this cluster prevents a correct trajectory analysis. From the calculated pseudotime, we have estimated the top 100 most variable genes along this lineage (**Figure 2D**). We can immediately notice that most of the genes that define the lineages towards cluster 2 and 3 are genes known to be involved in various immune responses such as an integrin, toll-like receptor, several cathepsins, or an antimicrobial peptide hydramacin. These gene sets reinforce the hypothesis that clusters 4 and 3 may represent two steps of hemocyte activation following a yet unknown signalling with the cluster 4 as an intermediate state and the cluster 3 as a terminal state of activation. Furthermore, for each of these clusters we are able to assign specific genes with a possible link to immune responses. A set of these genes alone can define each of the differentiated clusters in our analysis as cluster 2 (**Figure 2G**), cluster 3 (**Figure 2H**) and cluster 5 (**Figure 2I**).

### Use of the literature to define different transcriptomic clusters

The preliminary flow cytometry and microscopy approaches do not allow us to accurately connect morphotypes with scRNAseq transcriptomic clusters. We therefore relied on the literature to find marker genes for our different hemocyte populations. As the latter is currently very limited, we focused on the recent work of Li and collaborators (28) that analyse in *Biomphalaria* the transcriptomic expression of hyalinocytes and granulocytes. We selected immune genes that authors were able to extract from their analysis and that were more strongly expressed either by hyalinocytes or granulocytes. We retrieved those immune gene orthologs, indistinguishable from the two mollusk strains (BS-90 and M-line strains) studied, from each of the two morphological hemocyte populations. We then averaged the expression of all genes, by cell type, that we are able to detect in our scRNAseq analysis to determine which transcriptomic cluster would be very likely to express these immune gene pools (**Figures 2E, F**). The immune marker genes defined for hyalinocytes using this approach, do not fit a particular cluster for this cell morphotype. All the immune genes taken in this analysis are expressed in all the clusters (**Figure 2F**) and especially are not differentially expressed between the clusters (**Figure 2F**). On the other hand, and as expected from previous analyses, the immune genes expressed by granulocytes in the literature are found to be more expressed in a part of the cells of cluster 2 and in the majority of the cells of cluster 3 (**Figure 2E**). These genes defined as granulocyte-specific were also found to be slightly over-expressed in cluster 1. Furthermore, if we look at some specific genes, found in a few rare articles defining a gene for a probable hemocyte population in *Biomphalaria*, such as granulin (BgGRN: BGLB011796), suspected to be expressed by some granulocyte subtypes (28, 52, 53), we notice that this gene seems to evolve along the pseudotime to be expressed by clusters 2 and 3. A gene that might be associated with granulin would be a toll-like receptor (54) that is found in our analyses to be expressed also by cells of cluster 2 (**Supplementary Figure 3**). All of these evidences could lead us to believe that a specialization of hemocytes takes place from cluster 0 to clusters 4 and 3 or to cluster 2. The latter could then be associated with possible granulocytic activated cells. We also find the expression of a gene belonging to the Macrophage Migration Inhibitory Factor family (MIF: BGLB030407), this protein has been shown to be overexpressed in some granulocyte-like hemocytes (55) and could serve as regulating hemocyte, proliferation activation and encapsulation response. The gene coding for this protein in our analysis is not found in cluster 3 and expressed in all the other clusters and in particular with a gradient of expression between cells in cluster 2 (**Figure 2J**). We can speculate that this gene expression pattern could then be an activation sign of certain hemocytes and of differentiation towards granulocyte cells.

## Discussion

Our current knowledge about *Biomphalaria glabrata* hemocytes is still very sparse. The different populations of hemocytes have been only described so far by transmission electron microscopy (TEM) and flow cytochemistry approaches, or by optical microscopy and cell morphology description (24, 26, 27). Very few studies also address the question of the cell lineage of these hemocytes and the characterization of the hematopoiesis, by the amoebocyte producing organ (APO) (56, 57). To date, the scientific community agrees on the presence of at least 3 morphological populations defined only by their behaviour toward plating: hyalinocytes, granulocytes and blast-like cells. However, it seems to be a greater complexity and plasticity within these three large populations, and this diversity is very dependent on the tools used to characterize them (microscopy, flow cytometry, scRNA-seq).

Moreover, even the name given for each population was inconsistent in the literature, this increases the complexity of description of these populations [i.e., large adherent cells were called sometimes hyalinocytes or granulocytes depending on the paper considered (24, 26)]. For us, when hemocytes are allowed to adhere to a plastic slide, it is immediately obvious that the non-granulocytes adhere much better to the support by forming long pseudopodia and are considered as hyalinocytes (**Figure 1A**). However, it can also be seen that there are cells that are morphologically very close to hyalinocytes with intracytoplasmic granulations, that could be considered as adherent granulocytes or granular hyalinocytes (**Figure 1B**). Thus, the characterization of hemocytes by microscopy is very dependent on the technique employed (TEM, light microscopy, staining regent used,) or the support used for plating (polystyrene, plastic or glass slides). Moreover, distinguishing those hemocyte populations was particularly complicated in flow cytometry when all hemocytes are in suspension, they all display a spherical shape resulting in a continuum of size and granularity. Nevertheless, we have counted these different populations in naive snails and found that the majority of hemocytes were hyalinocyte-like cells, then blast-like cells and finally granulocytes (**Figure 1E**). These data are fairly consistent with the proportions determined by (24).

The scRNA-seq technology brings *de-novo* and without *a-priori* gene enrichment and heterogeneity information from a complex cell suspension. It brings information as well about potential role and function of each hemocyte transcriptional population but can also determine a possible lineage relationship between all these cells. For this purpose, we decided to study circulating hemocytes of naive *B. glabrata* snail, considered as not having undergone any experimental infection. We identified 2 large groups (**Figure 1F**) based on their transcriptomic identity, where a large and complex population of probable hyalinocyte (cluster 0, 2, 3, 4 and 5) is well separated from 2 distant populations (clusters 1 and 6) of differentiated hemocytes which could then be associated with probable blast-like cells, because of the few transcripts found in these two clusters (**Supplementary Figure 1**) or with granulocytes (**Figure 1F**). Moreover, the population distribution from microscopic observations fit the overall distribution of those 3 clusters (**Figure 1E**) (**Figure 1F**). All combined reveal a greater heterogeneity among circulating *B. glabrata* hemocytes compared to what have been defined by microscopy approaches alone (**Figure 1E**) but most likely potentially supported by a plasticity of hemocyte population (potentially from hyalinocyte activation pathway) rather than lineage-separated circulating cells. This high plasticity of hemocytes could also be accentuated by the large number of snails used to collect the hemocytes, which could add some inter-individual variation in the data analysis. This diversity in the origin of the hemocytes would then show the activation capacity of certain hemocyte types according to the specific physiological status of each individual, this could also explain the small number of cells observed for certain activated clusters such as cluster 3, which may originate from solely few individuals within the snails pool. The understanding of this plasticity rather than diversity of *B. glabrata* hemocytes will now deserve further investigation.

For each of these clusters, we provided a cell identity map by identifying their particular gene markers (**Supplementary Table 3**) to which we selected at least 5 marker genes defined as highly specific to each transcriptomic cluster (**Supplementary Table 1**). These marker genes helped us to define specific functions expressed by each of these hemocyte transcriptomic populations. However, the lack of deep annotation and literature for specific hemocyte markers prevent us from defining with precision a link between transcriptomic profile and morphological identity. At first glance, we hypothesised that hemocyte heterogeneity may be supported by plasticity in activation from few populations rather than large diversity of functionalized populations.

In an attempt to connect morphological features and scRNAseq clusters, we used FACS approaches of cell sorting based on basic morphological features such as size and granularity (FSC and SSC) of the hemocytes before analysing them with a global label-free proteomic approach. Nonetheless, as previously shown (47), *B. glabrata* hemocytes formed a continuum of size and granularity that does not allow us to accurately sort any particular hemocyte population. However, by defining specific gates on the FACS it was possible to recover fractions enriched by the three populations commonly accepted in our model (**Supplementary Figure 2**). From these sorted cell fractions, we performed a protein extraction, which was subjected to label-free LC-MS analyses. But unfortunately, the genes coding for the identified proteins did not allow us to link the hemocyte morphotypes to the different transcriptomic clusters. Indeed, we do not find by any particular differential expression of the marker genes defined for the hyalinocyte and blast-like cell fractions. However, a certain pattern seems to emerge for the genes belonging to the granulocyte-enriched fraction, which seem to be slightly overexpressed in the cells of clusters 1 and 6. These data seem to be corroborated by different genes identified from the literature that were assigned to granulocyte clusters. Indeed, we find genes such as granulin, some toll-like receptors more strongly expressed in these clusters 1 and 6 or cell differentiation factors such as MIF gene (**Figure 2J**) which is practically no longer expressed in clusters 1 and 6 and these hemocytes follow a particular expression pattern along the pseudotime when we use the cluster 0 as starting point. Clusters 1 to 6 would therefore represent differentiating or differentiated cells. These hemocyte clusters are composed of rather active cells even if in these clusters the RNA count is low (**Supplementary Figure 1**). The marker gene set detected is however very specific to these clusters and strongly linked to various immune responses.

However, we decided to remove clusters 1 and 6 from the analyses because the genes expressed in these two clusters are very different from the other clusters. It was then impossible to obtain a cell lineage between these two clusters and the rest of the clusters grouped around cluster 0. Either the cells that compose clusters 1 and 6 are from different progenitors or they are from different cell lines that we were not able to capture by this drop-seq approach. To solve this problem one possibility would be to increase the number of hemocytes captured in order to detect the rare hemocytes in our study model that could link all the transcriptomic clusters in the analysis. With this increase in the number of hemocytes captured, it would also be important to increase the depth and sensitivity of sequencing in order to be able to detect more genes in all cells like the use of well-based scRNAseq methods including smartseq2-3 (58, 59). For example in this analysis, we were not able to detect certain genes known to be expressed in hemocytes such as biomphalysin or BgTEP (30, 60). BgTEP were known to be expressed by a subpopulation of blast-like cells (29), thus using BgTEP as a marker gene would be particularly relevant, if we were able to detect it in scRNAseq approach.

Cluster 0 regroups the majority of the cells in our analysis and preferentially expresses a multitude of PRRs and genes involved in energy production. The cells of this cluster may be probably be considered as latent, sentinel cells, harboring a large diversity of receptors waiting to be activated by a contact with a stimulus (pathogen, circulating alarmins, etc). The cells in this cluster also express all the marker genes of the three hemocyte morphotypes defined on the basis of data from proteomics or the literature. It would therefore seem that this cluster is possibly composed of several different hemocyte morphotypes or only hyalinocytes cells due to the large number of genes expressed which would suggest large cells, waiting for a biotic or abiotic signal to engage in a specific differentiation pathway. One possible scenario would be that some of these cells, from cluster 0, would enter into division (which could represent cluster 4) and then produce active cells (represented here by the cells of cluster 3 linked in the pseudo-time to cells of cluster 4). This differentiation from cluster 0 to cluster 4 and cluster 3, may also be supported by the analysis of the MIF gene (**Figure 2J**) which could be considered as a differentiation factor towards granulocytes because of the decreasing of its expression along the pseudotime (**Figure 2J**) in the clusters identified in differentiation. This hypothesis of a progressive hemocyte differentiation may also be supported by recent studies conducted on crayfish (61) showing that in crustaceans a greater plasticity of hemocytes is also find and that the majority of hemocytes in this model are derived from a single common lineage. These hemocytes therefore tend to differentiate into functionally different populations where granulocyte-like hemocytes would be the terminal stage of differentiation. Based on our results, can we propose that a similar process of cell activation occurs in *B. glabrata* snails or more largely in invertebrates? This question will deserve further investigations.

One way to confirm this hypothesis would be to experimentally expose snails, and therefore hemocytes, to various pathogens and see if new populations would be able to emerge from this cluster 0. However, it is interesting to note that we find in cluster 6 and cluster 5, some sets of marker genes involved in responses to pathogens such as bacteria (cluster 5) or virus (cluster 6). These clusters could represent hemocytes that can be involved in the control of microbiota communities. Indeed, it is yet hypothesized that the immune system is necessary for the control and maintenance of the microbial communities associated with the holobionts in *B. glabrata* snails. This is particularly true for bacteria (62, 63) and potentially for virus control (64) in which clusters 5 and 6 may potentially be involved.

**Figure 3:**
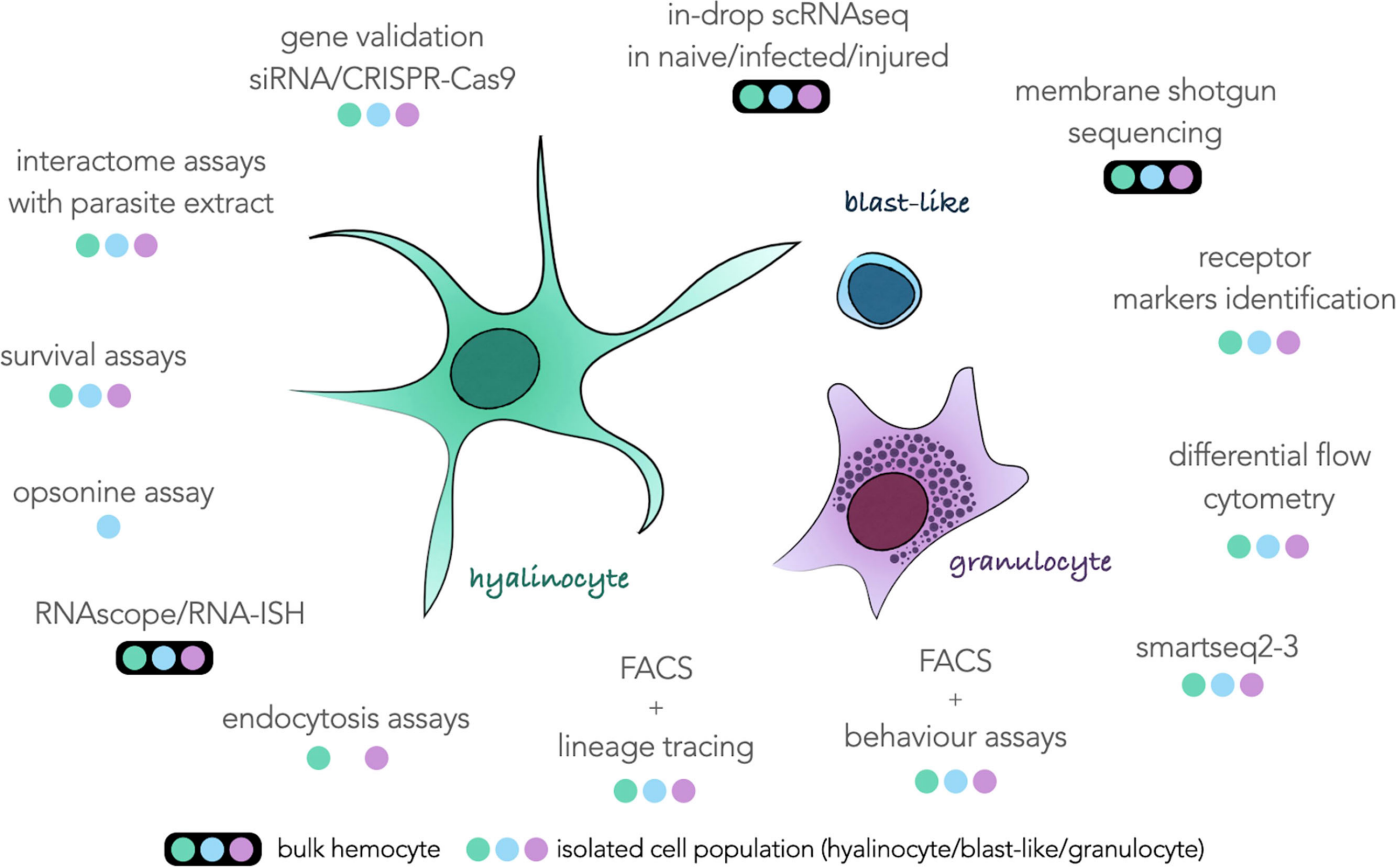
Schematic representation of the three hemocyte populations commonly described surrounded by all the techniques feasible on this type of cell and taking into account either bulk hemocytes (colored dots circled in black) or hemocytes taken individually (colored dots) according to their morphological characteristics. The three colored dots refer to the colors of each hemocyte population in the center of the figure.

All the results in this study still prevent us from assigning each transcriptomic cluster to a particular hemocyte morphotype with certainty. However, this scRNAseq analysis provides the basis for further descriptions of the function of each hemocyte type. The marker genes defined in this analysis allow us to draw an identity map of each of the transcriptomic clusters, and will be a terrific tool that can be used in a near future for the morphological validation of each of these cell groups. For example, a gene coding for a transmembrane protein (transmembrane 97-like) is highly expressed in cluster 1 and could be of great interest to determine if this cluster corresponds to one or more morphological cell types. But how can we physically separate our hemocytes? How to define more precisely the cells of cluster 0? The next challenges concerning the description of these hemocytes will consist in developing and combining more cutting-edge technology approaches of physical separation of hemocytes. We have summarized in **Figure 3** an exhaustive list of all the techniques and analysis that will need to be performed on these hemocytes to reveal their possible functions in the organism and the morphology associated with each of these functions.

These approaches can be split in two strategies, using hemocytes and complex tissues as a bulk or using single isolated cells. More in-drop isolated scRNAseq sequencing would bring new information about response to infection or injury in hemocytes. More sequencing would bring more accurate marker genes that could be used for targeted approaches such as *in-situ* hybridisation, siRNA or CRISPR-Cas9. Unfortunately, those approaches could still not be relevant to obtain membrane markers that could be used for FACS purposes. Indeed, surface markers often have low levels of RNA abundance and can be under the method sensitivity threshold. For this purpose, we propose to enrich hemocyte membrane proteins for MS-MS analysis to identify proteins harbored on hemocyte surfaces. Those proteins can then be used as targets for antibody-based isolation (FACS, MACS) or immunohistochemistry. As soon as several sub-populations of hemocyte can be isolated in flow cytometry gates, a novel era may start for the *Biomphalaria glabrata* community working on immunity. Obviously, it would pave the way for basic flow-cytometry immunology where cell populations are monitored following an immune stress, alarmin injection or parasite infection. The improvement of FACS techniques, coupled with approaches of more sensitive scRNAseq technologies such as smart-seq methods would increase the quality of transcriptome obtained for each population and be useful to characterize each of the morphological populations and their behaviour to certain pathogens or parasites. Moreover, those sorted sub-populations could then be used for *in-vitro* endocytosis or opsonin assays where we may observe the ability of the different hemocytes to interact, induce phagocytosis or actively endocyte differentially treated beads, parasites or pathogens.

Finally, determining the links between the different morphological populations and their functions is an essential step in understanding snail immune mechanisms, particularly in the context of immune response and innate immune memory acquisition towards the parasite *S. mansoni* responsible for intestinal schistosomiasis in humans. Much evidence leads to hemocyte while suggesting a support for innate immune memory. Here, we bring new evidence proving the plasticity over the diversity of hemocytes population in *Biomphalaria glabrata* and we bring new guidelines to transform hemocyte immunology research in invertebrates.

## Data availability statement

The datasets presented in this study can be found in online repositories. The data presented in the study are deposited in the Sequence Read Archive (SRA) repository, accession number PRJNA844512.

## Author contributions

Investigation and experimental procedures: RP, MP, AP, RG and BG. Bioinformatic analysis: RP, SP, CC. Writing—Original Draft Preparation: RP, SP and BG. Writing—Review & Editing: RP, SP, EV, DD, RG, and BG. Project. Administration: RG and BG. Funding Acquisition: DD and BG. All authors contributed to the article and approved the submitted version.

## Funding

This study is set within the framework of the “Laboratoires d’Excellences (LABEX)” TULIP (ANR‐10‐LABX‐41). DD and BG were supported by the ANR AEROSNAIL (ANR-19-CE11-0016-01) from the French National Research Agency (ANR). BG was supported by the BQR from the University of Perpignan. All authors contributed to the article and approved the submitted version.

## Acknowledgments

Thanks to cytometry facilities of the MRI platform (Montpellier ressource imagerie) as well as the EDyP service platform for their help in the development and implementation of cell sorting and label-free proteomics. Thanks to the technical staff of the IHPE laboratory, Jean-Francois Allienne for his expertise in molecular biology, Anne Rognon and Damien Pouzol for the maintenance of mollusc strains and their expertise on animal experimentation.

## Conflict of interest

The authors declare that the research was conducted in the absence of any commercial or financial relationships that could be construed as a potential conflict of interest.

## Publisher’s note

All claims expressed in this article are solely those of the authors and do not necessarily represent those of their affiliated organizations, or those of the publisher, the editors and the reviewers. Any product that may be evaluated in this article, or claim that may be made by its manufacturer, is not guaranteed or endorsed by the publisher.

## Supplementary material

Supplementary Figure 1 UMAP **(A)** and vlnplot **(B)** of RNA count by cell among the different transcriptomic clusters.

Supplementary Figure 2 Graph representing the distribution of occurrence by flow cytometry as a function of SSC and FSC measurement. The four colored windows noted from P1 to P4, correspond to the selection criteria of the occurrences to carry out the cellular sorting. The results of the microscopic observations of each of these fractions are noted on the right of the graph.

Supplementary Figure 3
**(A)** Expression of the granulin (BGLB011796) and **(B)** toll-like receptor (BGLB008602) gene along the pseudo-time and the representation of its expression by the different cells of the analysis on the UMAP representation.
