# Supplementary figures and images for: Single cell RNA sequencing reveals hemocyte heterogeneity in Biomphalaria glabrata: Plasticity over diversity
Source: Front Immunol. 2022 Sep 5;13:956871. doi: 10.3389/fimmu.2022.956871 (PMC9484523; doi:10.3389/fimmu.2022.956871)

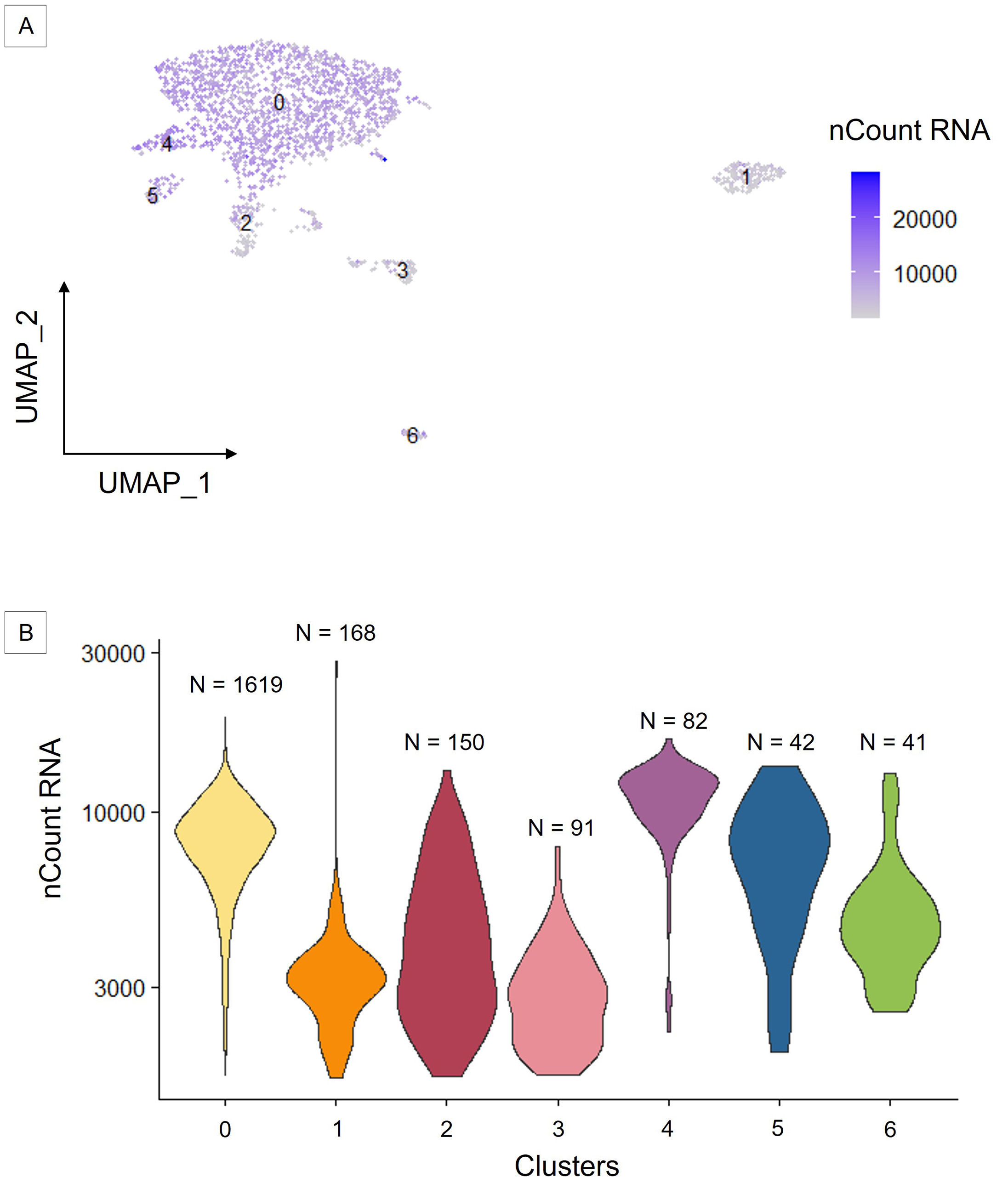

Supplement: Supplementary Figure 1 — UMAP (A) and vlnplot (B) of RNA count by cell among the different transcriptomic clusters. [file Image_1.jpeg]

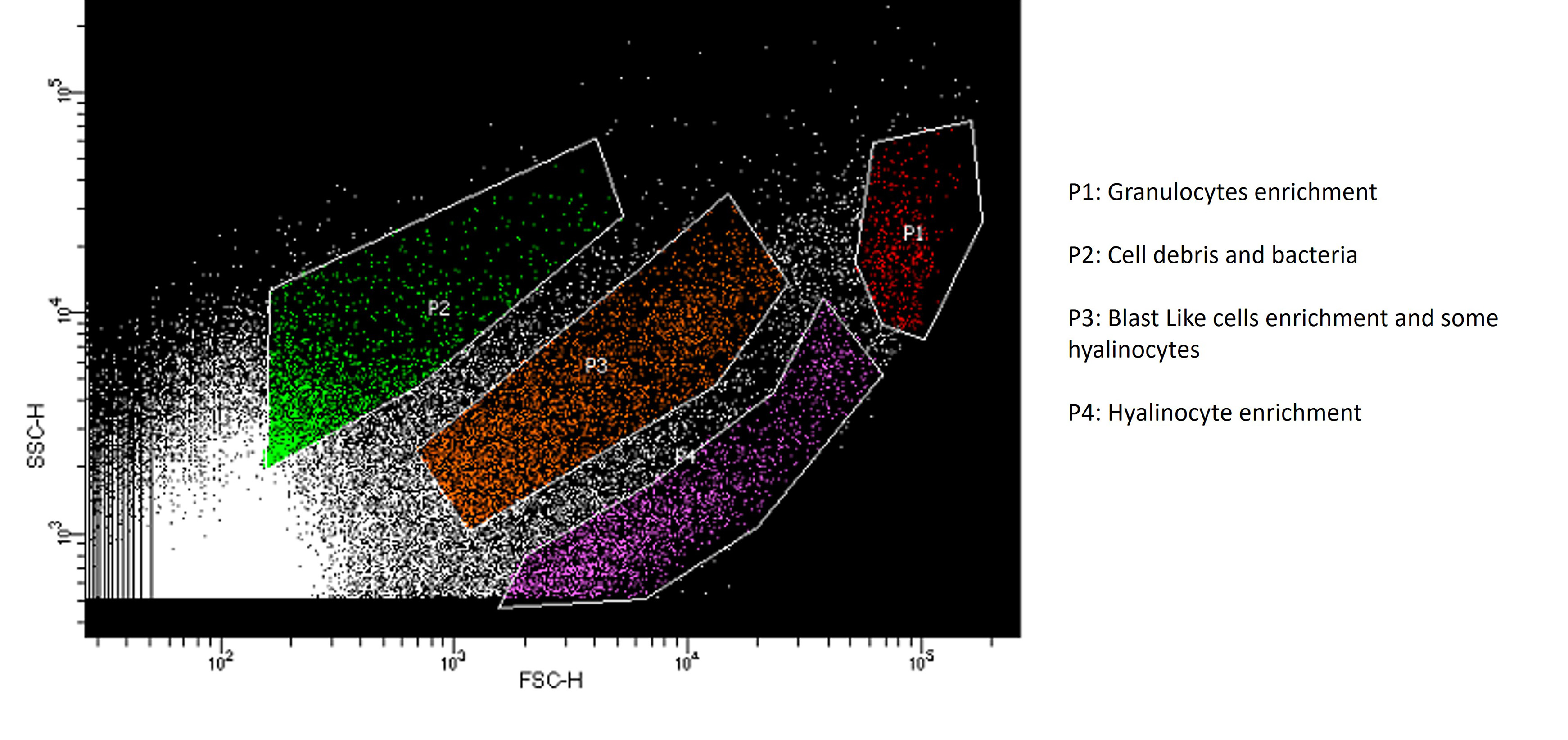

Supplement: Supplementary Figure 2 — Graph representing the distribution of occurrence by flow cytometry as a function of SSC and FSC measurement. The four colored windows noted from P1 to P4, correspond to the selection criteria of the occurrences to carry out the cellular sorting. The results of the microscopic observations of each of these fractions are noted on the right of the graph. [file Image_2.jpeg]

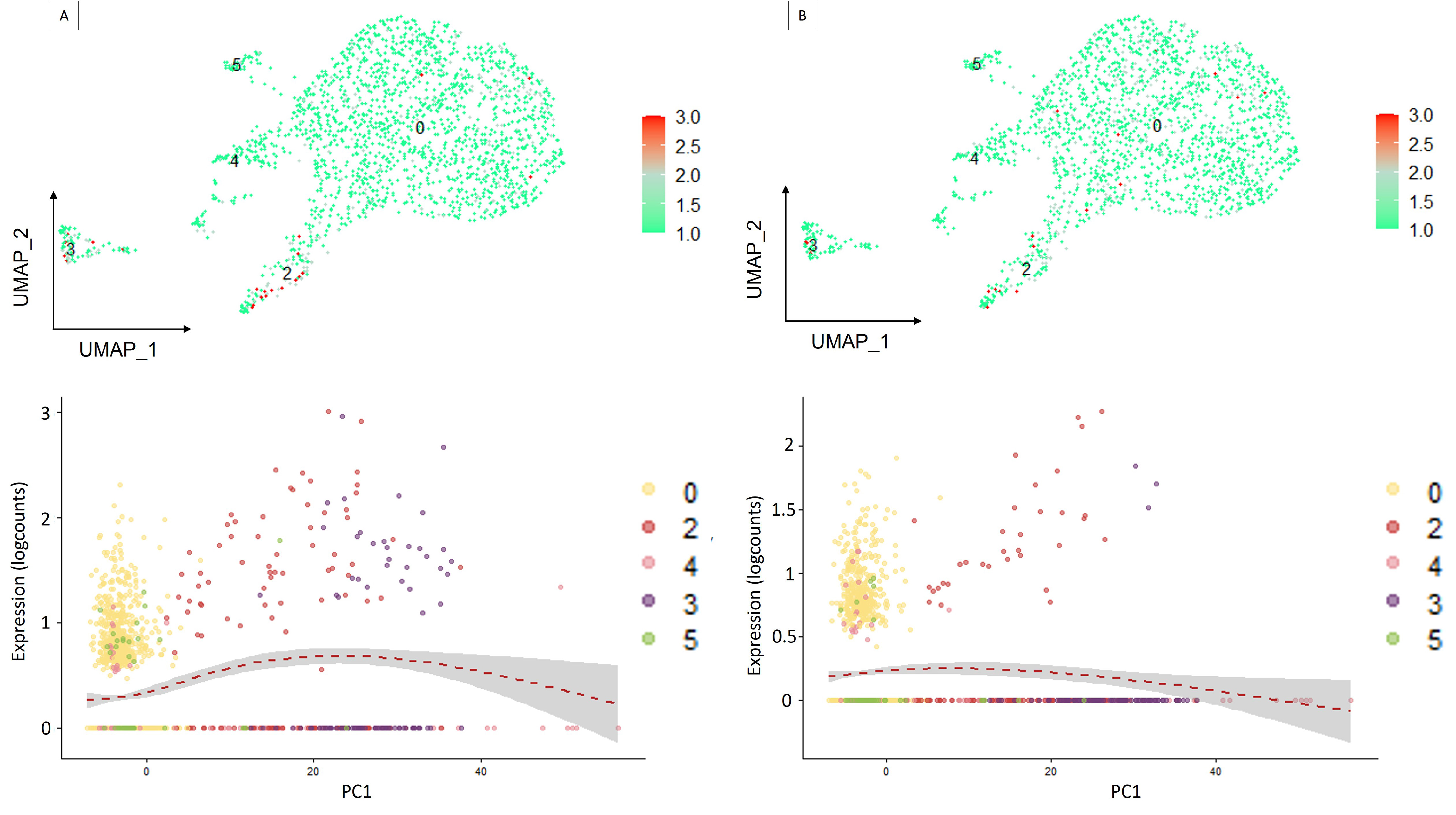

Supplement: Supplementary Figure 3 — (A) Expression of the granulin (BGLB011796) and (B) toll-like receptor (BGLB008602) gene along the pseudo-time and the representation of its expression by the different cells of the analysis on the UMAP representation. [file Image_3.jpeg]
